# Improving Virus Taxonomy by Recontextualizing Sequence-Based Classification with Biologically Relevant Data: the Case of the *Alphacoronavirus 1* Species

**DOI:** 10.1128/mSphereDirect.00463-17

**Published:** 2018-01-03

**Authors:** Gary R. Whittaker, Nicole M. André, Jean Kaoru Millet

**Affiliations:** a Department of Microbiology and Immunology, College of Veterinary Medicine, Cornell University, Ithaca, New York, USA; Icahn School of Medicine at Mount Sinai; Erasmus MC; Texas A&M University-Texarkana

**Keywords:** alphacoronavirus, *Alphacoronavirus 1*, biotype, canine coronavirus, clade, classification, coronavirus, feline coronavirus, serotype, transmissible gastroenteritis virus

## Abstract

The difficulties related to virus taxonomy have been amplified by recent advances in next-generation sequencing and metagenomics, prompting the field to revisit the question of what constitutes a useful viral classification. Here, taking a challenging classification found in coronaviruses, we argue that consideration of biological properties in addition to sequence-based demarcations is critical for generating useful taxonomy that recapitulates complex evolutionary histories. Within the *Alphacoronavirus* genus, the *Alphacoronavirus 1* species encompasses several biologically distinct viruses. We carried out functionally based phylogenetic analysis, centered on the spike gene, which encodes the main surface antigen and primary driver of tropism and pathogenesis. Within the *Alphacoronavirus 1* species, we identify clade A (encompassing serotype I feline coronavirus [FCoV] and canine coronavirus [CCoV]) and clade B (grouping serotype II FCoV and CCoV and transmissible gastroenteritis virus [TGEV]-like viruses). We propose this clade designation, along with the newly proposed *Alphacoronavirus 2* species, as an improved way to classify the *Alphacoronavirus* genus.

**IMPORTANCE** Our work focuses on improving the classification of the *Alphacoronavirus* genus. The *Alphacoronavirus 1* species groups viruses of veterinary importance that infect distinct mammalian hosts and includes canine and feline coronaviruses and transmissible gastroenteritis virus. It is the prototype species of the *Alphacoronavirus* genus; however, it encompasses biologically distinct viruses. To better characterize this prototypical species, we performed phylogenetic analyses based on the sequences of the spike protein, one of the main determinants of tropism and pathogenesis, and reveal the existence of two subgroups or clades that fit with previously established serotype demarcations. We propose a new clade designation to better classify *Alphacoronavirus 1* members.

## Abstract (precis)

Our work focuses on improving the classification of the *Alphacoronavirus* genus. The *Alphacoronavirus 1* species groups viruses of veterinary importance that infect distinct mammalian hosts and includes canine and feline coronaviruses and transmissible gastroenteritis virus. It is the prototype species of the *Alphacoronavirus* genus; however, it encompasses biologically distinct viruses. To better characterize this prototypical species, we performed phylogenetic analyses based on the sequences of the spike protein, one of the main determinants of tropism and pathogenesis, and reveal the existence of two subgroups or clades that fit with previously established serotype demarcations. We propose a new clade designation to better classify *Alphacoronavirus 1* members.

## INTRODUCTION

Viruses pose a classification conundrum because of the staggering size of the virome (1). This is particularly problematic for RNA viruses because of their high mutation rates, propensity to undergo recombination and/or reassortment, and the establishment of viral quasispecies upon infection. Recent advancements in metagenomics and next-generation viral genomic sequencing have exacerbated the problem because of the magnitude of new viral sequences discovered in a multitude of hosts and environmental samples. This has altered dramatically the known viral sequence landscape. To cope with the volume of new viral sequence data, it has been proposed that the International Committee on Taxonomy of Viruses (ICTV) include official classification of viruses based solely on virus genome and metagenomics sequence information (1).

These changes have prompted the field to revisit the question of what constitutes a useful viral classification. In this article, we focus on the *Alphacoronavirus 1* species of the *Alphacoronavirus* genus, to highlight some issues and limitations with current classification schemes and to provide some suggestions to improve them. This type species groups strains infecting distinct hosts and is based on a threshold level of more than 90% sequence identity in key coronavirus (CoV) replicase domains (pp1ab polyprotein and ORF1ab gene). However, this grouping leads to an association of viruses with divergent biological properties. Here, we propose a classification within the *Alphacoronavirus 1* species based on the spike (S) gene sequence that allows the recapitulation of the complex evolutionary histories.

Members of the *Coronaviridae* family form a diverse group of enveloped, single-strand, positive-sense RNA viruses. The *Coronaviridae* family is divided into the *Torovirinae* and *Coronavirinae* subfamilies, both characterized by their exceptionally large RNA genomes: 20.2 kb (*Bovine nidovirus* TCH5) to 33.5 kb (*Ball python nidovirus*) and 25.4 kb (*Porcine deltacoronavirus* HKU15) to 31.8 kb (*Bottlenose dolphin coronavirus* HKU22), respectively. *Torovirinae* and *Coronavirinae* subfamilies are able to infect a diverse array of vertebrate species. They have a distinct genomic architecture and replication strategy shared with other members of the *Nidovirales* order, which also includes the *Arteriviridae*, *Mesoniviridae*, and *Roniviridae* families (2, 3). Coronaviruses (CoVs) are classified into four genera, with *Alphacoronavirus* and *Betacoronavirus* containing members that infect mostly mammalian species and *Gammacoronavirus* and *Deltacoronavirus* grouping viruses infecting both birds and mammals (4).

The *Alphacoronavirus* genus is composed of viruses infecting bats, ferrets, mink, cats, dogs, pigs, and humans. The *Alphacoronavirus 1* type species is composed of the following prototypical viruses (5): feline coronavirus (FCoV), canine coronavirus (CCoV), and transmissible gastroenteritis virus (TGEV). FCoV is of particular interest as it manifests as two distinct biotypes (pathotypes) with a highly transmissible form, feline enteric coronavirus (FECV), which provokes self-limiting, usually mild, enteric tract infections, and a systemic form, feline infectious peritonitis virus (FIPV), typically associated with low transmissibility but high morbidity (6). In the widely accepted “internal mutation” hypothesis, it is believed that genetic mutations in the genome of FCoV occur within an infected animal, giving rise to FIPV (7). A similar FIP-like pathogenesis is also observed with ferret coronaviruses (FRCoVs) (8). While CCoV is a widespread enteric virus of dogs and can occur in highly pathogenic forms, the virus does not manifest itself with FIP-like clinical signs (9).

The coronavirus spike (S) envelope glycoprotein, the main determinant of virus entry, is an essential structural protein as it is the main surface antigen, governs binding to the host cell receptor, and mediates viral membrane fusion. S is typically primed proteolytically by host cell proteases to activate its fusogenicity (10, 11). As such, the coronavirus S protein is a critical component as it determines to a large extent host species, tissue, and cell tropism as well as pathogenicity and transmission. Previous serological characterizations of alphacoronaviruses, based on the antigenicity of the S glycoprotein, have revealed the existence of two distinct FCoV serotypes (serotypes I and II) (12–14). Both serotypes can manifest as either FECV or FIPV biotypes. FCoV serotype I is more prevalent in cats than serotype II but has proved more difficult to culture *in vitro* (15, 16). Likewise, for CCoV, two serotypes (I and II) have been characterized and are distinguished by genetic differences in S and ORF3 genes. Serotype II CCoV strains can be further subdivided into the IIa, IIb, and IIc subtypes (9). CCoV IIa and IIb strains are distinguished by differences in the N-terminal domain of the S protein (NTD), where the IIb NTD is closely related to the TGEV NTD. The recently characterized IIc subtype of CCoV has been reported in Sweden and in the United States.

The evolution of strains within the *Alphacoronavirus 1* species is complex and likely involved a number of recombination events. It is thought that serotype I FCoV and CCoV originated from a common ancestor. A recombination event occurring between a serotype I CCoV and an unknown coronavirus gave rise to serotype II CCoV, which acquired a recombinant S protein, distinct from serotype I S. TGEV appears to have originated from a serotype II CCoV (17). Additional, independent recombination events between serotype I FCoV and serotype II CCoV gave rise to serotype II FCoVs, such as FIPV-WSU-79-1146 and FECV-WSU-79-1683, which acquired a serotype II CCoV S protein (18). Furthermore, we have previously shown that CCoV strain A76 also has a recombinant S protein, a product of recombination between serotype I and II CCoV sequences (19). CCoV-A76 S was shown to have a serotype I-like S1 N-terminal domain (NTD), while the rest of the protein was serotype II-like. Analysis of coronavirus recombination events within the S protein sequence revealed its modular nature, allowing exchange of functional domains between coinfecting viruses (19, 20).

Because of the numerous recombination events occurring within the S gene of *Alphacoronavirus 1* species, current classification of *Alphacoronavirus 1* strains, based on key domains of the replicase polyprotein, fails to recapitulate previously established serotype demarcations. In addition to serological differences, serotype I and II S proteins are fundamentally different in several biological aspects. While the receptor for serotype II FCoV, CCoV, and TGEV has been shown to be aminopeptidase N (APN, or CD13), the receptor for serotype I strains remains unknown. Serotype I S proteins contain a cleavage site, the S1/S2 site, not present in serotype II or other alphacoronaviruses. This site has been shown to be important for cell culture adaptation and pathogenesis of FCoV (21, 22). Because of the critical role played by S protein in virus entry, pathogenesis, and tropism and since the S proteins of serotype I and II strains differ greatly, we propose a classification of *Alphacoronavirus 1* strains into two clades (*Alphacoronavirus 1* clades A and B), using a functionally based S protein sequence classification that reflects the previously determined serologically based demarcation. We argue that, when available, combining function-based data with sequence-defined taxonomic groupings should be encouraged as it allows for useful, biologically relevant virus classifications.

## RESULTS

**FIG 1:**
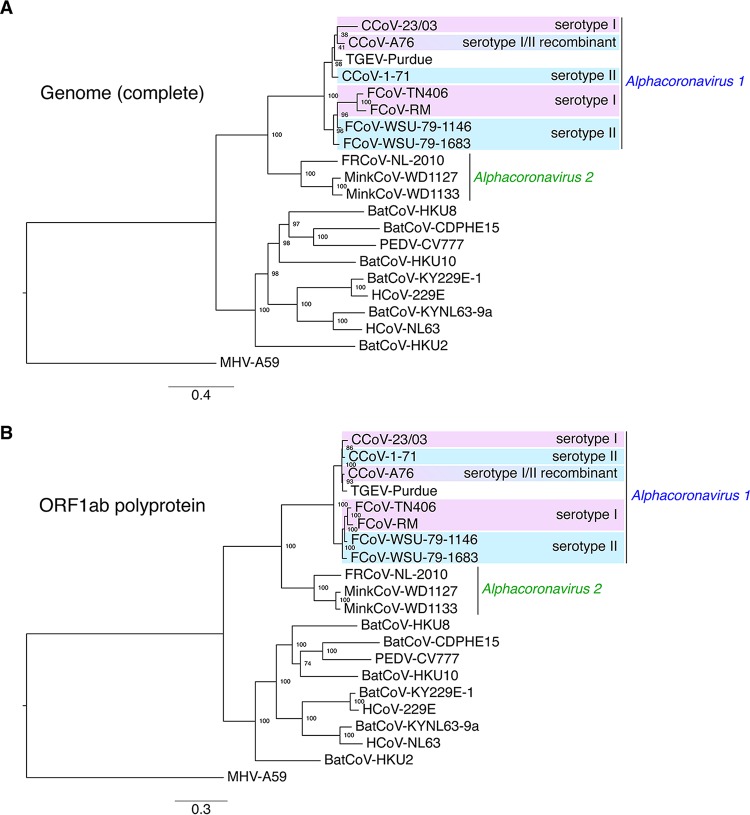
Phylogenetic analyses of alphacoronaviruses based on complete genome and ORF1ab protein sequence. Nucleotide sequences of the complete genomes of alphacoronaviruses and of the betacoronavirus MHV-A59 (A) or the complete protein sequences of the ORF1ab polyprotein of the corresponding viruses (B) were aligned using MAFFT within the Geneious 10 software package. The alignments were then used to generate maximum-likelihood phylogenetic trees using PhyML (25). The trees were rooted with MHV-A59. Numbers at nodes indicate the bootstrap support on 100 replicates. The scale bar indicates the estimated number of substitutions per site. Accession numbers for complete genome nucleotide sequences and ORF1ab protein sequences used are found in Materials and Methods.

As a starting point to gain a better understating of the phylogenetic relationships between alphacoronaviruses, we generated a phylogenetic tree of key representative species and strains based on complete genome nucleotide sequence alignment (Fig. 1A). As expected, the *Alphacoronavirus 1* FCoV, CCoV, and TGEV strains formed a well-defined monophyletic group. The analysis also reveals the clearly delineated branching of coronavirus strains that infect ferrets and mink, FrCoV-NL-2010, MinkCoV-WD1127, and MinkCoV-WD1133, which were recently proposed to form a separate species, *Alphacoronavirus 2* (23) (Fig. 1A). Two main subgroupings within the *Alphacoronavirus 1* species were observed, with CCoV and TGEV partitioning into one subgroup and FCoV forming a second, separate subgroup. The complete genome-based phylogenetic tree clusters CCoV and FCoV strains according to their respective host species.

Analysis based on the ORF1ab polyprotein sequence reveals very similar phylogenetic relationships, with a partitioning of FCoV strains in one subgroup and CCoV and TGEV strains in another (Fig. 1B). Similarly to the complete genome analysis, the ORF1ab polyprotein-based phylogenetic groups strains according to host species.

Both complete genome and ORF1ab polyprotein cases fail to recapitulate the biologically relevant antigenic demarcations. In particular, the CCoV-A76 recombinant strain clusters with other serotype II CCoVs and TGEVs in both trees. However, we have previously shown that this strain harbors a recombinant S protein that has a distinct antigenic profile compared to other serotype II CCoVs, such as the CCoV-1-71 prototype strain, as it showed no reactivity to any of the serotype II-specific monoclonal antibodies tested (19). The whole-genome and ORF1ab phylogenetic tree analyses again show their limitations, as they fail to discern the unique biological properties of the CCoV-A76 strain.

**FIG 2:**
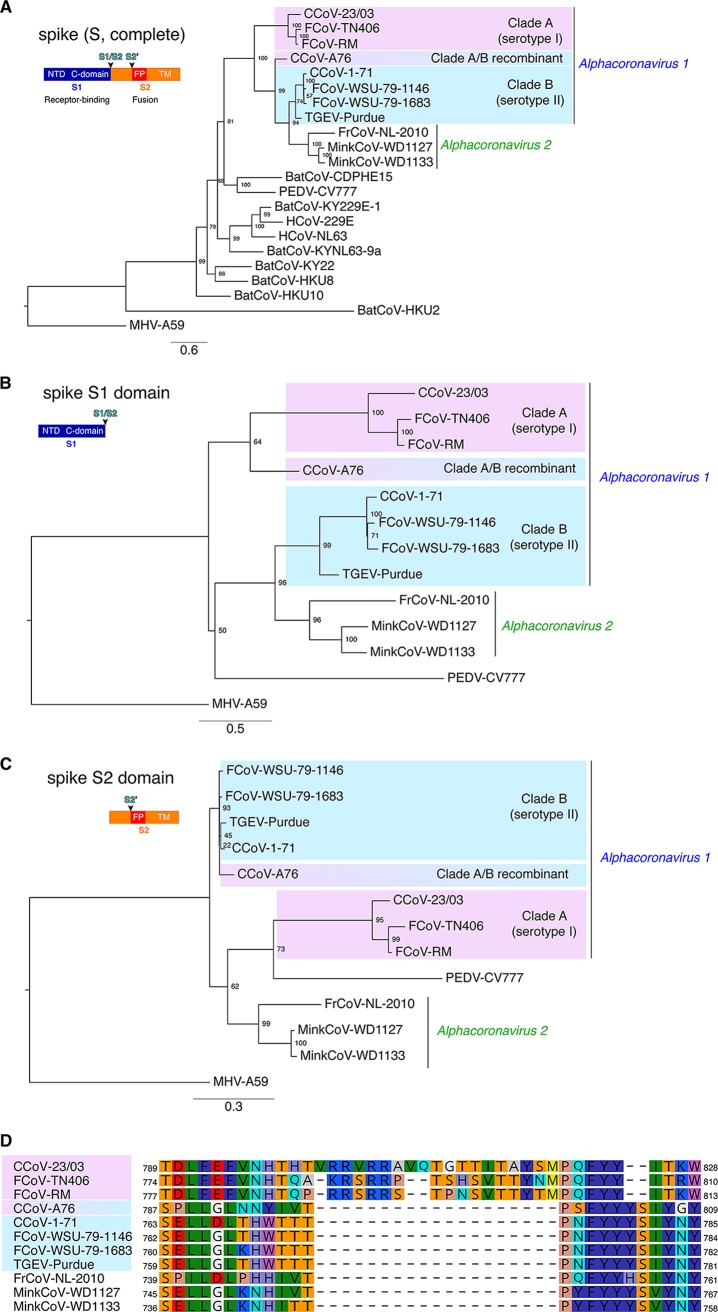
S protein sequence-based phylogenetic analyses of alphacoronaviruses. (A to C) Protein sequences of the complete S protein (A), S1 domain (B), and S2 domain (C) were aligned using MAFFT within the Geneious 10 software package, and maximum-likelihood phylogenetic trees were generated with PhyML. The trees were rooted using MHV-A59. Numbers at nodes indicate the bootstrap support on 100 replicates. Scale bars indicate estimated numbers of substitutions per site. (D) The sequences corresponding to the S protein region around the S1/S2 cleavage site of *Alphacoronavirus 1* strains and closely related viruses were aligned with MAFFT using the Geneious 10 software package. For all panels, accession numbers for spike (S) protein sequences used are found in Materials and Methods. NTD, N-terminal domain; C-domain, C-terminal domain; FP, fusion peptide; TM, transmembrane domain.

In contrast, when a phylogenetic analysis based on full-length S protein alignment is performed (Fig. 2A), a different partitioning of *Alphacoronavirus 1* strains is revealed. Serotype I FCoV and CCoV cluster in one group, and serotype II FCoV, CCoV and TGEV are grouped in another. Furthermore, the CCoV-A76 strain is found at an intermediate position between the two serotypes (19), a result that reflects the recombinant nature of its S protein. This phylogenetic analysis clearly demarcates strains according to the previously characterized serotypes and not according to which host species the strains infect, as was observed when performing the analysis with complete-genome or ORF1ab sequences.

Since the coronavirus S protein S1 and S2 are functionally distinct domains, with S1 involved in receptor binding and being the main target of neutralizing antibodies while S2 governs viral fusion, we further analyzed these domains separately using *Alphacoronavirus 1* strains and closely related species (Fig. 2B and C). The S1 analysis shows similar relationships as in the complete spike analysis but with the recombinant CCoV-A76 strain being more closely related to clade A viruses, reflecting its serotype I-derived NTD (Fig. 2A and B) (19). For the S2 analysis, while the serological demarcations are retained, the CCoV-A76 strain is found more closely related to clade B or serotype II strains due to its recombinant S protein (Fig. 2C). A characteristic feature of serotype I FCoV and CCoV is an S cleavage site containing a polybasic furin recognition motif at the junction between the S1 receptor-binding domain and the S2 fusion domain, S1/S2 (Fig. 2D). Alignment of protein sequences of alphacoronavirus S1/S2 S cleavage sites reveals that serotype I CCoV and FCoV contain a 16- to 19-amino-acid insert, with a stretch of basic residues, flanked by fairly well conserved N- and C-terminal regions. This feature is absent in serotype II FCoV, CCoV, TGEV, and ferret CoV, mink CoV, or other alphacoronaviruses. The lack of an S1/S2 furin site in CCoV-A76 S protein sequence is in agreement with the fact that only the NTD of the CCoV-A76 S protein is serotype I-like, with the rest of the protein being serotype II-like, including the region around the S1/S2 site. Overall, these alignment observations are in good agreement with the complete S, S1, and S2 phylogenetic tree analyses.

Using such S-based analyses, we propose that the *Alphacoronavirus 1* species be subclassified as clade A, corresponding to serotype I FCoV and CCoV, and clade B, corresponding to serotype II FCoV and CCoV and TGEV-like viruses.

## DISCUSSION

The field of virus taxonomy is undergoing major changes due to the massive increase in viral sequence data obtained through next-generation sequencing and metagenomics efforts. The field is in need of novel methodologies to classify viruses, which are currently being implemented by the ICTV (1). Proposed methods allow for the establishment of classification based on sequence data alone. We argue that when available, it is still important to incorporate or add biological/phenotypic data when establishing virus classification, as sequence-based demarcations can in some instances lead to inconsistent groupings, exemplified here with the *Alphacoronavirus 1* type species.

Current classification within the *Alphacoronavirus 1* species is not well defined and often fails to recognize the profound differences observed between well-established *Alphacoronavirus 1* serotypes. Adding to the confusion are the different terms used to designate various *Alphacoronavirus 1* strains: FCoV serotypes and types; CCoV serotypes, types, and genotypes; and TGEV, which is not classified according to FCoV/CCoV serotypes. We propose a more unified classification, based on the important serological and sequence differences between the S proteins of serotype I and II viruses. Our analysis reveals two well-defined clades, clade A and clade B, corresponding to the serotype groupings. Both clades contain FCoV and CCoV strains, while TGEV belongs only to clade B, in agreement with the finding that TGEV is most closely related to clade B (serotype II) CCoV. We recommend the inclusion of representatives of both clades when performing phylogenetic analysis of alphacoronaviruses. The proposed classification scheme for the alphacoronaviruses is similar to the one used to characterize lineages and clades of avian influenza viruses. Indeed, instead of performing phylogenetic analyses on the entire genomes, avian influenza virus classifications are based on the surface protein genes, e.g., that for hemagglutinin (HA) (24).

In addition to better matching serological and phylogenetic groupings, S-based phylogenies offer other advantages. Because the S gene is frequently shuffled by recombination events, such classifications allow the grouping of viruses that have a shared S gene. In our analyses, using complete genome phylogenies, strains were clustered according to the host that they infected, whereas the S-based phylogeny allowed grouping of FCoV and CCoV strains together in separate clades. Our approach allows for a better understanding of the complex phylogenetic relationships and evolutionary history observed in coronaviruses. Furthermore, phylogenetic analysis on S proteins can reveal relationships that are not observed using replicase domains or complete-genome-based analysis. In particular, in a study characterizing novel deltacoronaviruses, Woo and colleagues showed that the S proteins of alphacoronaviruses are more closely related to deltacoronaviruses than to other coronavirus genera (4).

Our alignment analysis of the S1/S2 cleavage site of alphacoronaviruses and its agreement with phylogenetic analyses suggest that the presence of a furin motif consisting of a stretch of basic residues only at the S1/S2 site of clade A viruses could be used for rapid determination of clade inclusion in samples which are difficult to sequence at a whole-genome level.

While current criteria based on analyses of key replicase domains allow the definition of coronavirus species, we believe that it is useful to also provide subspecies classifications based on other genetic loci such as the S gene to allow for more functionally relevant classifications.

This work highlights the importance of incorporating biological data into sequence-based classifications for virus taxonomy. Within the *Alphacoronavirus 1* species, this allows for more biologically relevant and useful virus groupings. We believe that this practice should continue to be encouraged and/or added once such data become available for viruses discovered solely through sequence information.

## MATERIALS AND METHODS

### Sequence information.

To perform genomic phylogenetic analyses, the following nucleotide sequences of the complete genomes of representative alphacoronaviruses and the betacoronavirus mouse hepatitis virus (MHV)-A59 were retrieved from GenBank (NCBI accession numbers in parentheses): CCoV-23/03 (KP849472.1), CCoV-A76 (JN856008.2), TGEV-Purdue (AJ271965.2), CCoV-1-71 (JQ404409.1), FCoV-TN406 (EU186072.1), FCoV-RM (FJ938051.1), FCoV-WSU-79-1146 (NC_002306.3), FCoV-WSU-79-1683 (JN634064.1), FRCoV-NL-2010 (NC_030292.1), MinkCoV-WD1127 (HM245925.1), MinkCoV-WD1133 (HM245926.1), BatCoV-HKU8 (NC_010438.1), BatCoV-CDPHE15 (NC_022103.1), porcine epidemic diarrhea virus (PEDV)-CV777 (AF353511.1), BatCoV-HKU10 (JQ989270.1), BatCoV-KY229E-1 (KY073747.1), human coronavirus (HCoV)-229E (KU291448.1), BatCoV-KYNL63-9a (NC_032107.1), HCoV-NL63 (AY567487.2), BatCoV-HKU2 (NC_009988.1), and MHV-A59 (AY700211.1).

For analyses using the ORF1ab protein, the following sequences were retrieved: CCoV-23/03 (AKZ66481.1), CCoV-1-71 (AFG19735.1), CCoV-A76 (AEQ61967.2), TGEV-Purdue (P0C6Y5.1), FCoV-TN406 (ABX60144.1), FCoV-RM (ACT10853.1), FCoV-WSU-79-1146 (YP_004070193.2), FCoV-WSU-79-1683 (AFH58022.1), FRCoV-NL-2010 (YP_009256195.1), MinkCoV-WD1127 (ADI80512.1), MinkCoV-WD1133 (ADI80522.1), BatCoV-HKU8 (ACA52170.1), BatCoV-CDHPE15 (AGT21332.1), PEDV-CV777 (P0C6Y4.1), BatCoV-HKU10 (AFU92103.1), BatCoV-KY229E-1 (APD51497.1), HCoV-229E (AOG74782.1), BatCoV-KYNL63-9a (YP_009328933.1), HCoV-NL63 (AAS58176.2), BatCoV-HKU2 (ABQ57207.1), and MHV-A59 (AAU06353.1).

To perform phylogenetic analyses based on the spike (S) protein, the following S protein sequences were used: CCoV-23/03 (AAP72150.1), FCoV-TN406 (BAC05493.1), FCoV-RM (ACT10854.1), CCoV-A76 (AEQ61968.1), CCoV-1-71 (AAV65515.1), FCoV-WSU-79-1146 (YP_004070194.1), FCoV-WSU-79-1683 (AFH58021.1), TGEV-Purdue (ABG89335.1), FRCoV-NL-2010 (AKG92640.1), MinkCoV-WD1127 (ADI80513.1), MinkCoV-WD1133 (ADI80523.1), BatCoV-CDHPE15 (AGT21333.1), PEDV-CV777 (AAK38656.1), BatCoV-KY229E-1 (APD51499.1), HCoV-229E (BAL45637.1), HCoV-NL63 (AAS58177.1), BatCoV-KYNL63-9a (YP_009328935.1), BatCoV-KY22 (ADX59495.1), BatCoV-HKU8 (ACA52171.1), BatCoV-HKU10 (AFU92104.1), BatCoV-HKU2 (ABQ57208.1), and MHV-A59 (AAA46455.1).

### Phylogenetic analyses.

The above-mentioned nucleotide or protein sequences were aligned with Multiple Alignment using Fast Fourier Transform (MAFFT; https://mafft.cbrc.jp/alignment/software/) within the Geneious 10 software package (Biomatters, Auckland, New Zealand). For each coronavirus species, S protein sequence alignments enabled us to determine the demarcation between S1 and S2 domains based on the position of the S1/S2 cleavage site found in some alphacoronaviruses. PhyML was used to generate maximum-likelihood (ML) trees based on whole-genome nucleotide, ORF1ab protein, and S protein complete and partial (S1 and S2 domain) alignments. Whole-genome, ORF1ab protein, and S protein sequences of the betacoronavirus MHV-A59 were used for rooting phylogenetic trees. Bootstrap support was calculated from 100 replicates.
